# Production of IgY polyclonal antibody against diphtheria toxin and evaluation of its neutralization effect by Vero cell assay

**DOI:** 10.1186/s12896-021-00694-7

**Published:** 2021-05-12

**Authors:** Morteza Rezaeifard, Roya Solhi, Mohammad Mohammadi, Ebrahim Abbasi, Mahdi Aminian

**Affiliations:** 1 Department of Clinical Biochemistry, School of Medicine, Tehran University of Medical Sciences, Poursina Street, Keshavarz Boulevard, Tehran, Iran; 2 Department of Clinical Biochemistry, Faculty of Medical Sciences, Tarbiat Modares University, Tehran, Iran; 3 Department of Biochemistry, School of Medicine, Hamedan University of Medical Science, Hamedan, Iran; 4 Department of Bacterial Vaccines, Razi Vaccine and Serum Research Institute, Karaj, Iran; 5 Recombinant Vaccine Research Center, Tehran University of Medical Sciences, Tehran, Iran

**Keywords:** Diphtheria toxin, IgY, Purification, Vero cell

## Abstract

**Background:**

Diphtheria is a bacterial disease which is caused by *Corynebacterium diphtheriae*. The symptoms are due to the diphtheria toxin produced by the bacteria. Antibiotic therapy and the use of diphtheria antitoxin is a recommended strategy to control diphtheria. Although mammalian antibodies are used to treat patients, IgY antibody has advantages over mammalian ones, including cost-effectiveness and production through non-invasive means. Moreover, in contrast to mammalian antibodies, IgY does not bind to the rheumatoid factor and does not activate the complement system. The objective of this study was to evaluate the in vitro neutralizing effect of IgY against diphtheria toxin.

**Results:**

Anti-DT IgY was produced by immunization of the laying white leghorn chickens. Indirect enzyme-linked immunosorbent assay revealed successful immunization of the animals, and the IgY was purified with a purity of 93% via polyethylene glycol precipitation method. The neutralizing activity of the purified IgY was evaluated by Vero cell viability assay. This assay confirmed that 1.95 μg (8.6 μg/ml of culture medium) of anti-DT IgY would neutralize 10 fold of cytotoxic dose 99% of DT, which was 0.3 ng (1.33 ng/ml of culture medium).

**Conclusion:**

This anti-DT IgY may be applicable for diphtheria treatment and quality controls in vaccine production.

**Supplementary Information:**

The online version contains supplementary material available at 10.1186/s12896-021-00694-7.

## Background

Diphtheria is an infectious disease caused by *Corynebacterium diphtheria*. It is often localized in the upper respiratory tract, which produces a thick and grey pseudomembrane. Diphtheria leads to difficulty in breathing, swallowing, myocardial complications, and finally, brain anoxia which can cause death [1]. The bacteria secrete a protein called diphtheria toxin (DT), which is about 62 kDa consists of 535 amino acids. Diphtheria toxin plays a principal role in diphtheria pathogenesis in which the protein synthesis is arrested by ADP ribosylation of elongation factor 2 [2].

Because of the high rate of immunization, diphtheria incidence has declined dramatically. However, due to low vaccination coverage in some countries, loss of vaccine-induced immunity over time, increases in international travels, and the emergence of new biotypes, diphtheria has not been eradicated in the world [3]. According to World Health Organization, more than 7000 cases of diphtheria were reported worldwide in 2016 [4]. Moreover, fatal cases have also occurred over the last decade among developing and developed countries throughout the world [5].

The recommended strategy for controlling diphtheria infection is antibiotic therapy in combination with diphtheria antitoxin (DAT) [6]. Antibiotic treatment after manifestation of the symptoms may not be effective enough. Hence, Finding an appropriate antibiotic for the treatment of multi-drug resistant strains of the bacteria is a considerable problem [7]. For a long time, equine-derived DAT was applied to provide patients with passive immunity. Serum sickness associated with horse-derived DAT is a drawback of its efficacy [6, 8]. Antibodies from immunized human individuals are also used for the treatment of the patients. However, in addition to the disease transfer problem, the supply of human derived-serum is limited, and the production of DAT from the immunized population is not cost-effective [5, 9]. The risk of serum-borne diseases, hypersensitivity, the growing needs, and extremely limited resources altogether provide an incentive to find more efficient alternatives for current diphtheria antitoxins [10].

IgY antibodies have received plenty of attention due to several advantages, including low cost and non-invasive means of production compared to mammalian antibodies [11, 12]. Moreover, IgY does not react with mammalian complement and rheumatoid factors (RF) [13, 14]. RF is an autoantibody that binds to the Fc region of mammalian IgG, causing interference in many immunoassays [15]. In contrast to mammalian antibodies, IgY does not bind to the cell surface Fc receptors and provides higher stability against environmental conditions such as pH and temperature [12, 16]. Interestingly, several researchers have demonstrated that IgY antibodies can be used for therapeutic applications, especially in infectious diseases and toxin neutralization [16–20].

Nevertheless, to the best of our knowledge, there has been no report on the efficacy of IgY polyclonal antibody against diphtheria toxin. This study aims to produce anti-DT IgY and evaluate its neutralization effect against cell toxicity of diphtheria toxin.

## Results

### ELISA titration of anti-DTx IgY

**Fig. 1:**
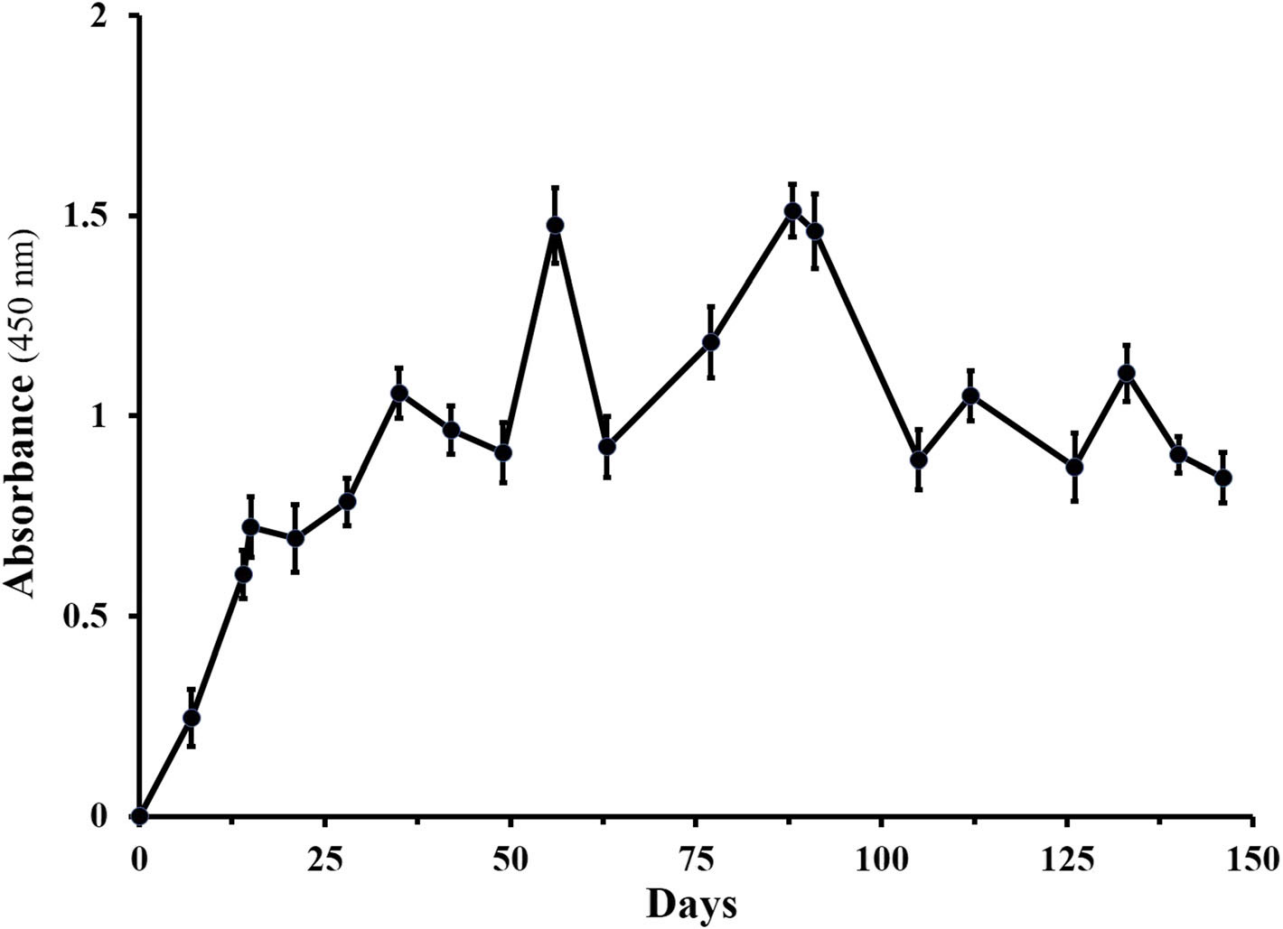
Titration curve of IgY production. A group of two chickens was immunized with 0.1 mg of diphtheria toxoid on days of 0, 18, 43, and 62. The level of IgY was determined by indirect ELISA using diphtheria toxoid. Each experiment was performed in two replicates, and the mean ± SD of samples was represented. All Data are normalized against the mean value of the control group injected by PBS and adjuvant

Levels of specific anti-diphtheria toxoid IgY were measured by ELISA (Fig. 1). Increases in specific IgY titers were observed after each injection and reached the highest amounts after the fourth immunization. The IgY titers remained relatively high after the last booster for 5 months, with some variations. There was no increase in the titer of specific IgY in the control group. After the second immunization, eggs were collected daily, and the yolks were pooled for the subsequent purification.

### IgY purification

**Fig. 2:**
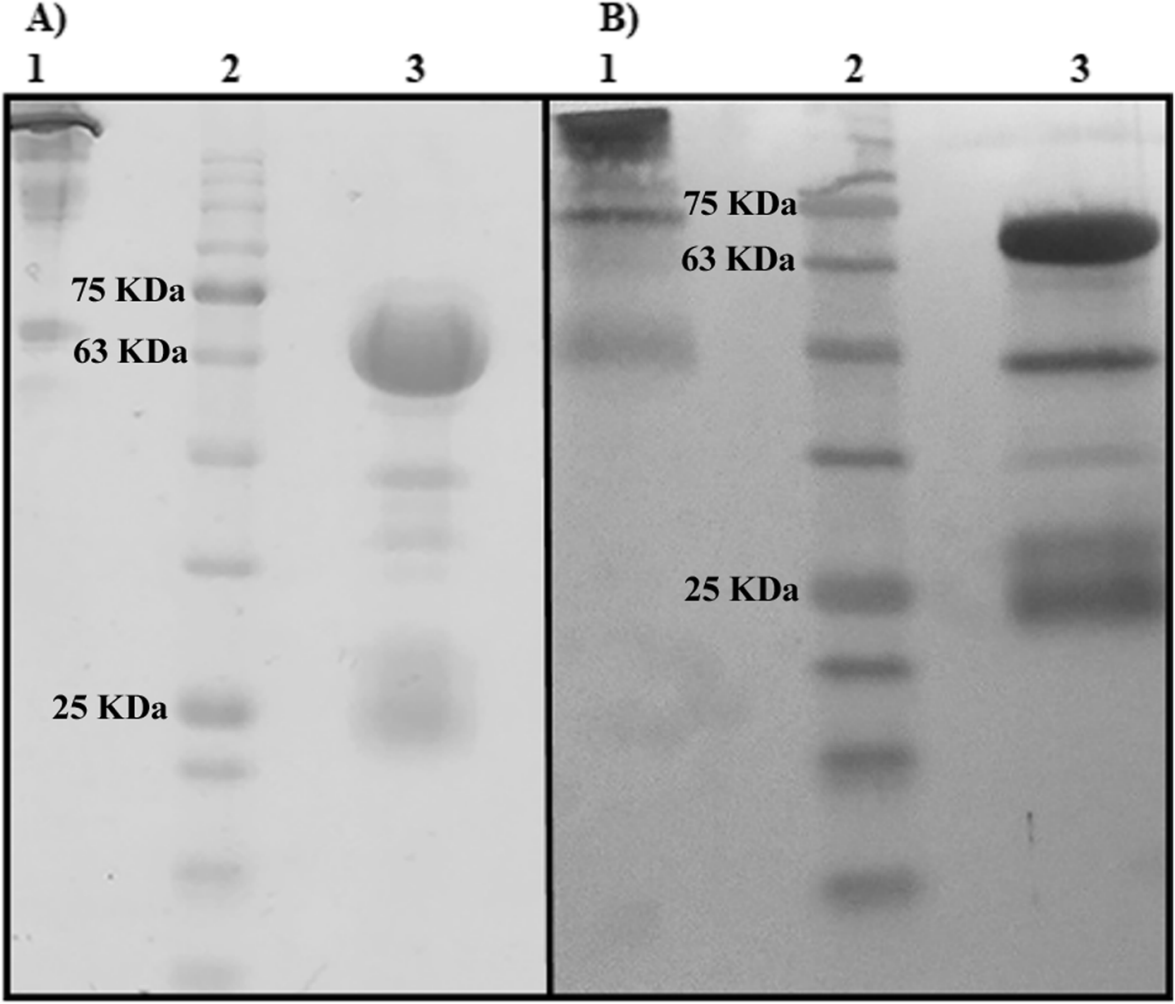
Analysis of the PEG purified anti-DT IgY. **a** SDS-PAGE analysis. The IgY samples were resolved on 12% gel and stained by Coomassie Brilliant Blue R-250. **b** Western blot analysis. The IgY samples were run on a 12% SDS-PAGE gel and transferred onto a nitrocellulose membrane. The samples were probed by peroxidase-conjugated rabbit anti-chicken IgY. 1: Non-reducing condition. 2. Molecular weight marker. 3: Reducing condition

Purification of IgY from egg yolks performed by polyethylene glycol (PEG) precipitation method and the sodium dodecyl sulfate-polyacrylamide gel electrophoresis (SDS-PAGE) results are demonstrated in Fig. 2. The ImageJ analysis verified a purity of about 93%. Purified IgY showed a protein band with the apparent molecular weight of 180 kDa under non-reducing conditions. Under reducing conditions, it was resolved into 67 and 22 kDa protein bands related to heavy and light chains of the antibody, respectively.

### Western blot analysis

Western blot analysis was performed to confirm the protein bands, which were considered IgY by SDS-PAGE. HRP conjugated anti-chicken IgY reacted strongly with 180 kDa protein band under non-reducing conditions. The conjugated anti-chicken IgY also detected the protein bands of heavy and light chains with 67 and 22 kDa molecular weight under reducing conditions, respectively.

### The cytotoxic dose of DT

The cell viability assay was used to quantify the cytotoxicity of diphtheria toxin. The cytotoxic dose of 99% of the toxin on Vero cells was calculated about 0.03 ng (0.13 ng/ml of culture medium) by taking the highest dilution of DT in which the color of the medium has not been changed. In the control group, the color of the medium was turned to yellow following cell growth.

### Neutralization activity of anti-DT IgY

In the DT neutralization assay, anti-DT IgY was diluted twofold and was challenged with 10 MCD of the toxin. Neutralization activity of anti-DT IgY was assayed by pH color change test in the microplate. The lowest quantity of IgY, which neutralized 10 CD99 of the toxin was considered as effective dose 99% (ED99) and was calculated to be 1.95 μg (8.6 μg/ml of culture medium) of purified IgY. The ED99 value of equine antitoxin (positive control) was determined to be 1.56 μg (6.9 μg/ml of culture medium). In the negative control group, in which toxin was treated with PBS-immunized IgY, cells were not grown, and the color of the medium remained red. In the toxin control group, Vero cells were treated with 10 CD99 of toxin, and the color of the medium remained red, indicating the total cell death.

## Discussion

Diphtheria remains endemic in several developing countries, even following comprehensive vaccination programs [3, 10, 21–23]. Conventional equine diphtheria antitoxin is the current treatment for the neutralization of Diphtheria toxin [6]. However, due to limited supplies and the problems related to serum sickness, finding an alternative is highly considerable [8, 10, 24–26].

The IgY enjoys several prominent advantages described previously [11–14]. Several studies are demonstrating the neutralizing effect of egg yolk-derived immunoglobulin (IgY) against different toxins, pathogens, and diseases [16, 27–31]. However, there is no report of the neutralization effect of IgY on diphtheria toxin to date. In the present study, the chickens were immunized with diphtheria toxoid, and the DT-specific IgY was purified using the PEG precipitation method. The SDS-PAGE analysis of purified IgY showed a purity of about 93%. It was shown that purification of IgY via PEG precipitation is a preferred method compared to the other purification methods using dextran or chloroform chemicals [32]. Akita and ‘Nakai’s study demonstrated IgY purity of about 96% using the PEG purification method [33].. Considering the Akita and Nakai study results, our results show acceptable purity (93%).

In this study, the neutralizing effect of anti-DT IgY was evaluated against diphtheria toxin using Vero cell viability assay. For potency estimation of antibodies against diphtheria toxin, Vero cell viability assay is approved as an equivalent to Guinea pig in-vivo assay by WHO (WHO 2005) [34]. This assay revealed that the amount of effective dose 99% (ED99) of anti-DT IgY was 1.95 μg (8.6 μg/ml of culture medium), which would effectively neutralize 10 fold of CD99 of the toxin. This result shows the ability of DT-specific IgY to neutralize the cell toxicity of diphtheria toxin. Considering the amount of ED99, DT-specific IgY shows reasonable and acceptable results of neutralization activity against the cell toxicity of diphtheria toxin in comparison with equine diphtheria antitoxin, 1.56 μg (6.9 μg/ml of culture medium).

Kanchana Usuwanthim et al. developed two murine monoclonal antibodies (mAbs) that could neutralize DT in Vero cell assay. The effective dose of 50% (ED50) of the mAbs to neutralize 10 Cytotoxic doses 50% (CD50) of DT (0.084 ng) was 50 ng and 160 ng [35]. Considering that only a portion (%5–10) of a whole IgY is specific for an antigen and comparing the concepts of CD50 to CD99 as well as ED50 to ED99, our results could be comparable to the results of their study.

In another study, an anti-DT monoclonal antibody was made by Leila M. Sevigny et al. using recombinant technology. They found that 5.5 ng/ml of this monoclonal antibody (315C4) can achieve total neutralization of DT using a Vero cell-based assay [36]. The protective effect of this monoclonal antibody is very strong and far from other results. Because of insufficient information on their Vero cell assay, we cannot compare the results of anti-DT IgY to their monoclonal antibody. Like other polyclonal antibodies, anti-DT IgY is a mixture of antibodies recognizing different specific epitopes. Moreover, the production of IgY has the advantage of being much more rapid, less expensive, and less technical skill than is required to produce MAbs [37].

## Conclusion

The successful results of our study and also the cost and limited supply of commercial anti-DT antibodies suggest that anti-DT IgY could be used as an alternative antibody against diphtheria toxin. Our findings promote further complimentary evaluation of the neutralization effect of anti-DT IgY under in vivo conditions. Furthermore, this anti-DT IgY may be applicable for in-process and final quality controls in vaccine production as an alternative to equine antibodies.

## Methods

### Diphtheria toxin and toxoid

Diphtheria toxin (DT) and toxoid (DTx) were obtained from Razi Vaccine and Serum Research Institute (Karaj, Iran), lyophilized to concentrate the proteins, and stored at − 20 °C. The concentrations of samples were determined by the Bradford method [38].

### Animals

The 24-week old laying white leghorn chickens weighing 1200 to 1500 g were obtained commercially from a local poultry farm (Tehran, Iran), prepared, and kept in individual cages. Chickens had been vaccinated against common poultry diseases and had access to food and water ad libitum. This study was reviewed and approved by the Ethics Committee of the Tehran University of Medical Sciences (No 24271–284541).

### Immunization schedule

A pair of chickens were immunized intramuscularly at 4 sites in the breast muscle with 0.1 mg of diphtheria toxoid in 0.5 ml of phosphate-buffered saline (PBS, pH 7.4), which were emulsified with an equal volume of complete ‘Freund’s adjuvant (CFA). Another pair of chickens received emulsified PBS with CFA and were used as control. Animals were randomized into the treatment group and control group by the complete randomization method. On days 18, 43 and 62, each chicken received booster injections with incomplete ‘Freund’s adjuvant (IFA) following the first injection. Eggs were collected daily for 5 months after the first injection and stored at 4 °C for the next steps. Finally, chickens were sacrificed by CO_2_ inhalation which was approved by the Ethics Committee of the Tehran University of Medical Sciences.

### Determination of IgY-DTx levels

An indirect ELISA was used to evaluate the antibody titers of eggs obtained from the immunized chickens. Each experiment was performed in two replicates, and the mean ± SD of samples was calculated by Excel software 2010. The yolks were separated and diluted (1:5 v/v) with distilled water, and pH was adjusted to 5 and incubated overnight at 4 °C. Then samples were centrifuged at 13000 g for 20 min at 4 °C, and the supernatant was stored at − 20 °C as water-soluble fractions (SWFs) [39]. The microplate wells (Nunc) were coated with 100 μl (20 μg/ml) of diphtheria toxoid in carbonate/bicarbonate buffer (0.1 M, pH: 9.6) overnight at 4 °C. Unbound toxoids were washed with PBS, and non-specific bindings were blocked with 250 μl/well of 2.5% skimmed milk in PBS buffer for 2 h at room temperature (RT). After washing 3 times with PBS, 100 μl of 1:2000 diluted WSF samples (immunized, non-immunized, and PBS) were added to wells and incubated for 2 h at room temperature. The microplate was washed thoroughly three times with PBS buffer. One hundred μl of diluted (1:32000) HRP-conjugated rabbit anti-chicken IgY (Sigma) in PBS/Tween 0.1% (PBST) was added and incubated for 2 h at room temperature. After washing 4 times with PBST, the color was developed by adding 100 μl substrate solution (1 mg of Tetramethylbenzidine in 157 μl of ethanol, 10 ml H_2_O, 1 ml of acetate buffer 1.1 M pH 5, and 2 μl of H_2_O_2_). After 30 min, the reaction was stopped with HCl 2 N, and the absorbance of wells was measured at 450 nm.

### IgY purification

IgY Polyclonal antibody was purified from immunized ‘chicken’s egg yolk with PEG method as described previously [40]. Briefly, the yolk was diluted (1:3 v/v) with PBS. For precipitation of lipid particles, 3.5% (w/v) PEG 6000 was added and mixed with a magnetic stirrer for 20 min at RT. The supernatant containing IgY was isolated by centrifugation (13,000 g for 20 min at 4 °C) and filtered through filter paper. Another 8.5% (w/v) of PEG 6000 was added to the supernatant and mixed for 20 min at RT. The mixture was then centrifuged (13,000 g for 20 min at 4 °C), and the pellet was dissolved in 10 mL PBS. 12% (w/v) of PEG 6000 was added to the solution and mixed for 20 min at RT, followed by centrifugation as above. The resulting pellet was dissolved in 5 ml of PBS, dialyzed overnight against 2 mM phosphate buffer (pH 6.5), and finally saved at − 20 °C.

### SDS-PAGE and Western blot analysis

The purity of IgY was evaluated by SDS-PAGE under reducing and non-reducing conditions. SDS-PAGE was carried out in 5% stacking gel (1.0 M Tris-HCl buffer pH 6.8) and in 12% separating gel (1.5 M Tris-HCl buffer pH 8.8) using a vertical slab gel apparatus [41]. IgY samples were treated with sample buffer containing 1% SDS, 0.001% bromophenol blue, 50 mM Tris– HCl (pH 6.8), 1% β-mercaptoethanol in reducing conditions, and without β-mercaptoethanol in non-reducing conditions. Then, samples in reducing conditions were heated for 5 min at 100 °C. After electrophoresis, the gels were stained with Coomassie brilliant blue R250 solution, and destaining steps were performed for visualization of protein bands. The gels were scanned and analyzed via densitometry using ImageJ software (1.48) to calculate the purity of IgY. Verification of IgY was performed by Western blot analysis [42]. Blotting was carried out by transferring the IgY bands from SDS-PAGE gels to the nitrocellulose membrane. The membrane was then blocked with 5% skimmed milk in PBS for 2 h at RT. Consequently, the peroxidase-conjugated rabbit anti-chicken IgY (Sigma,1:4000 dilutions) was added and incubated for another 2 h at RT. Finally, the protein bands were visualized by freshly prepared 3,3′-Diaminobenzidine (DAB) substrate solution (9 mg of DAB and 15 μl of 30% H_2_O_2_ in 20 ml of 50 mM Tris buffer, pH 7.4). Extensive washing was performed using PBST (0.1% Tween in PBS) following each step.

### Cell culture

Vero cells were obtained from Razi Vaccine and Serum Research Institute (Karaj, Iran). The cells were grown in 25 cm^2^ polystyrene tissue culture flasks with Dulbecco’s modified Eagle’s minimum essential medium (DMEM) supplemented with 10% fetal bovine serum (FBS), 1% penicillin (10.000 IU/ml) and streptomycin (10 mg/ml) at 37 °C in a humidified CO_2_ incubator. Vero cells were harvested by treating with 1 ml of 0.25% trypsin in PBS buffer containing 0.1% ethylenediaminetetraacetic acid (EDTA) and were suspended to a final density of 1 × 10^6^ cells/ml in fresh supplemented DMEM for further cell viability assays [43].

### Minimum cytotoxic dose of diphtheria toxin

DT is a cytotoxic protein for Vero cells. The cell viability assay was performed to quantify DT cytotoxicity using the color change method. Twenty-five μl of two-fold serial dilutions of DT were prepared in wells of tissue culture microplate using sterile PBS. Then, 150 μl of supplemented DMEM medium and 50 μl of Vero cell (5 × 10^4^ cells) were added to each well. The wells without toxin were considered as control. The tissue culture microplate was incubated for 4–5 days under 5% CO_2_ at 37 °C. In the wells in which the toxin inhibited the cellular metabolism, the color of the medium was not changed. The highest dilution of DT, in which the color of media was not changed, has been taken into account for the determination of cytotoxic dose of diphtheria toxin, which kills 99% (CD99) of the cells [44].

### Neutralization activity of IgY-DTx

Neutralizing activities of IgY-DTx against diphtheria toxin were evaluated by the Vero cell viability assay [44]. Twenty-five μl of two-fold serial dilutions of filter-sterilized purified IgY were prepared in wells of tissue culture microplate using sterile PBS. Then, 25 μl of filter-sterilized DT (10x of CD99) was added to each well and incubated for 60 min at 37 °C. After toxin neutralization, 125 μl of supplemented DMEM medium and 50 μl of Vero cell (5 × 10^4^ cells) were added to each well, and finally, the plate was incubated for 4–5 days under 5% CO_2_ at 37 °C. The effective dose of 99% (ED99) of IgY-DTx was calculated by taking the highest dilution of IgY at which the cells were grown, and the color of the medium changed to yellow. Two groups were considered as negative controls, one containing PBS-immunized IgY and the other one containing PBS without any IgY sample. Purified equine immunoglobulins diphtheria antitoxin (Razi vaccine and serum research institute, Karaj, Iran) was used in parallel neutralizing assay and assigned as a positive control.

## Supplementary Information

**Additional file 1:.**

## Data Availability

The datasets supporting the conclusions of this article are included within the article (and its additional files).

## Abbreviations

IgY: Immunoglobulin Y
DT: Diphtheria Toxin
DTx: Diphtheria Toxoid
DAT: Diphtheria AntiToxin
ELISA: Enzyme Linked Immuno-Sorbent Assay
PEG: Polyethylene Glycol
SDS-PAGE: Sodium Dodecyl Sulphate- PolyAcrylamide Gel Electrophoresis
HRP: HorseRadish Peroxidase
CD99: Cytotoxic Dose 99
ED99: Effective Dose 99%
RF: Rheumatoid Factors
CFA: Complete Freund’s Adjuvant
IFA: Incomplete Freund’s Adjuvant
WSFs: Water-Soluble Fractions
PBS: Phosphate Buffer Saline
PBST: Phosphate Buffer Saline Tween
DAB: DiAminoBenzidine
DMEM: Dulbecco’s Modified Eagle’s Minimum
EDTA: EthyleneDiamineTetraacetic Acid
RT: Room Temperature
MCD: Minimum Cytotoxic Dose
FBS: Fetal Bovine Serum
MAbs: Monoclonal AntiBodies
