# Supplementary material for: Production of IgY polyclonal antibody against diphtheria toxin and evaluation of its neutralization effect by Vero cell assay
Source: BMC Biotechnol. 2021 May 12;21:34. doi: 10.1186/s12896-021-00694-7 (PMC8117566; doi:10.1186/s12896-021-00694-7)
Supplement: Supplementary file 1 — Additional file 1:. [file 12896_2021_694_MOESM1_ESM.docx]

**Production of IgY polyclonal antibody against diphtheria toxin and evaluation of its neutralization effect by Vero cell assay**

Morteza Rezaeifard, Roya Solhi, Mohammad Mohammadi, Ebrahim Abbasi, Mahdi Aminian.

**Original picture of Fig. 2A**

N1 N2 1 2 3


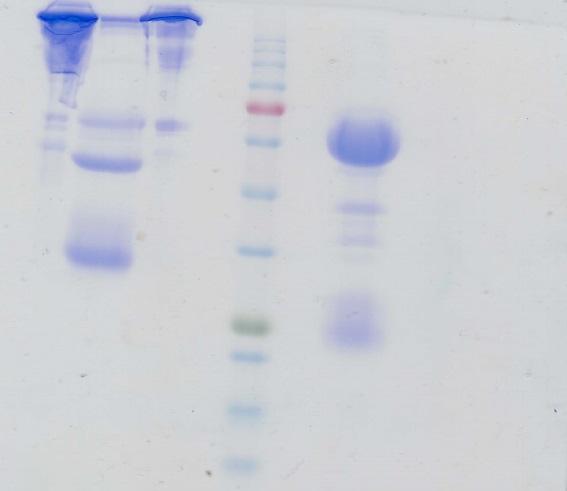


SDS-PAGE analysis. The IgY samples were analyzed on 12% gel and stained by Coomassie Brilliant Blue R-250. 1: Non-reducing condition. 2. Molecular weight marker. 3: Reducing condition. N: Non-used samples from purification steps (N1 and N2: non-reducing conditions).

**Original picture of Fig. 2B**

N1 1 2 3 N2


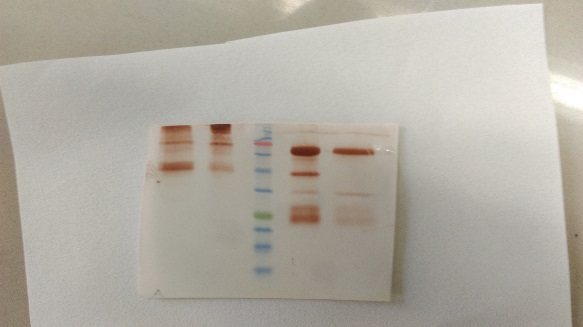


Western blot analysis. The IgY samples were run on a 12% SDS-PAGE gel and transferred onto a nitrocellulose membrane. The samples were probed by peroxidase-conjugated rabbit anti-chicken IgY. 1: Non-reducing condition. 2. Molecular weight marker. 3: Reducing condition. N: Non-used samples from purification steps (N1: non-reducing and N2: reducing conditions).
